# Neurological Complications and Viral Myositis in Pediatric Patients with Influenza Infection Attending a Tertiary Hospital in Madrid

**DOI:** 10.3390/microorganisms14102262

**Published:** 2026-10-05

**Authors:** Paula Cabrera Cascajero, Nieves Dueñas Sánchez-Valdemoro, Alessandra G. Villanueva Guerra, Guillermo F. Ruiz-Ocaña de las Cuevas, Marta Illán Ramos, Adrián García Ron, Arantxa Berzosa Sánchez, Eva Arias Vivas, José T. Ramos Amador

**Affiliations:** 1 Department of Pediatrics, Hospital Clínico San Carlos, Hospital Clínico San Carlos Health Research Institute (IdISSC), Centro de Investigación Biomédica en Red de Enfermedades Infecciosas (CIBERINFEC), 28040 Madrid, Spain; 2 Department of Pediatrics, Hospital Universitario 12 de Octubre, Instituto de Investigación Sanitaria (i+12), Centro de Investigación Biomédica en Red de Enfermedades Infecciosas (CIBERINFEC), Instituto de Salud Carlos III (ISCIII), Universidad Complutense de Madrid (UCM), 28041 Madrid, Spain

**Keywords:** influenza, children, neurological complications, seizures, encephalitis, myositis

## Abstract

Background: Influenza infection in children may be associated with neurological complications, including seizures and encephalitis, as well as suspected acute viral myositis, which may result in significant morbidity. Objective: This study aimed to estimate the incidence of neurological complications and suspected acute viral myositis in pediatric patients with laboratory-confirmed influenza and explore clinical and epidemiological characteristics potentially associated with these complications. Methods: A retrospective observational study was conducted including children aged 0–18 years with laboratory-confirmed influenza A or B who attended a tertiary hospital in Madrid between 2017 and 2023. Neurological complications were defined as seizures or encephalitis during the influenza episode. Incidence of complications and hospital admissions was analyzed across epidemiological periods (pre-pandemic, pandemic, and post-pandemic). Results: A total of 617 influenza episodes were identified. Neurological complications occurred in 33 patients (5.3%) and suspected acute viral myositis in 23 (3.7%). Seizures were the most frequent neurological manifestation (66.7%). Underlying neurological disease was strongly associated with neurological complications (31.6% vs. 3.6%; *p* < 0.01). Myositis was more frequent in influenza B infection (7.3% vs. 1.8%; *p* < 0.01). The frequency of neurological complications and suspected acute viral myositis did not differ significantly across epidemiological periods. Conclusions: Neurological complications and myositis were uncommon in pediatric influenza. Pre-existing neurological diseases were strongly associated with neurological complications.

## 1. Introduction

Influenza is a highly prevalent infection in the pediatric population and represents a significant public health concern each year.

Influenza is estimated to account for approximately 10% of all respiratory hospitalizations in individuals under 18 years of age worldwide [1]. In Spain, it remains a frequent cause of hospitalization in the pediatric population, particularly among infants and children with underlying conditions [2]. Therefore, annual universal vaccination in childhood is recommended [3].

Central nervous system involvement is a relatively common complication of influenza infection in the pediatric population, although it is poorly documented and likely underdiagnosed. It has been reported that among children hospitalized with influenza, between 7% and 10% develop neurological complications, with those with underlying neurological disorders being particularly vulnerable [1,4].

Influenza can be associated with a broad spectrum of neurological manifestations, ranging from mild symptoms, such as headache or syncope, to well-defined neurological complications. The most common include seizures and encephalitis, which generally have a favorable course but may be associated with clinically significant morbidity [4,5].

Within this spectrum, influenza-associated encephalitis represents one of the most severe forms. It typically occurs during the first days of the respiratory illness and is thought to be related to a dysregulated systemic proinflammatory response rather than direct viral invasion of the central nervous system [4,5]. In its most severe forms, it may be associated with brain lesions, high mortality, and neurological sequelae, as seen in acute necrotizing encephalopathy [6,7]. Its potential severity reinforces the indication for universal vaccination.

Another recognized manifestation in the pediatric population is acute myositis, which typically appears during the early days of infection and follows a self-limited course. It is known to occur more frequently in the context of influenza B infection and typically presents with muscle pain, difficulty walking, and elevated creatine kinase levels; in most cases, it is a benign condition without sequelae [8,9].

Despite advances in understanding these complications, their optimal management remains not fully established. Although most neurological and muscular manifestations are self-limited, some may result in significant morbidity. Identifying children at increased risk of neurological complications, particularly those with pre-existing neurological conditions, may facilitate early recognition and closer clinical monitoring.

The primary objective of this study was to estimate the incidence of neurological complications and acute viral myositis associated with influenza infection in pediatric patients treated at Hospital Clínico San Carlos during the study period. Secondary objectives were to describe the epidemiological characteristics of the study population and of children who developed neurological complications or myositis, explore clinical and epidemiological characteristics potentially associated with their occurrence, assess whether the SARS-CoV-2 pandemic modified their incidence, characterize coinfections identified in association with influenza as well as their potential relationship with neurological complications, and determine the morbidity and mortality associated with these complications.

## 2. Materials and Methods

An observational, descriptive, retrospective study was conducted.

All patients aged between 0 months and 18 years with laboratory-confirmed influenza A or B infection, diagnosed by polymerase chain reaction (PCR) or antigen testing, who attended the Emergency Department, pediatric wards, or Pediatric Intensive Care Unit at Hospital Clínico San Carlos between January 2017 and December 2023 were included. As a referral hospital, Hospital Clínico San Carlos recorded a mean of 11,824 pediatric emergency visits per year during the study period.

As this was a retrospective cohort with consecutive case inclusion, no prior sample size calculation was performed, and all identified patients were included.

All patients were retrospectively identified through ICD-10 (International Classification of Diseases, 10th revision) codes for diagnosis at discharge and chart review including only patients with a confirmatory laboratory diagnosis of influenza. Those patients without laboratory confirmation were excluded. Data abstraction was standardized according to a predefined data collection form. Both previously healthy patients and those with neurological disease or neurodevelopmental disorders were included, as one of the objectives of the study was to assess whether these conditions were associated with the development of neurological complications in the context of influenza infection. All patients with laboratory confirmation by viral antigen detection or PCR in respiratory secretions were included. Respiratory samples obtained by nasopharyngeal aspirate were processed for influenza A and B detection using commercial immunochromatographic assays for rapid antigen detection and/or real-time PCR (preceded by ribonucleic acid reverse transcription), according to the diagnostic testing performed during clinical care. Because of the retrospective design, the medical records did not consistently document the criteria used to select rapid antigen testing versus PCR, nor the manufacturer or analytical sensitivity of the rapid antigen assays.

Among hospitalized patients, respiratory viral coinfections were assessed using a nasopharyngeal multiplex PCR panel (FilmArray). Blood cultures were obtained when clinically indicated to assess for bacterial coinfection. Therefore, the microbiological assessment of coinfection was not performed using a uniform protocol across the entire cohort.

Clinical and microbiological data were retrospectively obtained from electronic medical records. Variables were entered into an anonymized database specifically designed for this study. Cases with only clinical suspicion of influenza without microbiological confirmation were excluded.

The primary study variables were the presence of neurological complications and suspected acute viral myositis during the influenza episode. Neurological complications were defined as seizures or acute encephalitis. Influenza-associated seizures were defined as any clinical or electrographic seizure occurring in a child without a known seizure disorder, or as an increase in the frequency of seizures above baseline in a child with a known seizure disorder. A diagnosis of acute encephalitis was based on a compatible clinical presentation, including altered mental status, confusion or irritability out of proportion to medical illness, supported by cerebrospinal fluid and/or electroencephalographic findings, when available; detection of influenza virus by PCR in cerebrospinal fluid was not required.

Suspected cases of acute viral myositis were defined by the presence of myalgia accompanied by elevated creatine kinase (CK) levels during influenza infection. CK values were recorded from the medical records.

Secondary variables of clinical and epidemiological interest were also collected, including relevant medical history, age, sex, influenza vaccination status, mild neurological symptoms (syncope, headache, and vertigo), presence of viral coinfections (such as SARS-CoV-2 or respiratory syncytial virus), suspected or confirmed bacterial superinfection, resolution of neurological symptoms, and treatments administered.

To evaluate the impact of the SARS-CoV-2 pandemic on the frequency of neurological complications, suspected acute viral myositis, and hospital admission rates, data were analyzed across three predefined calendar periods: pre-pandemic (2018–2019), pandemic (2020–2021), and post-pandemic/transition period (2022–2023). The number of pediatric emergency visits in each biennium was used as the denominator to account for differences in the overall volume of pediatric emergency care across periods and to allow a comparable assessment of the frequency of influenza-associated complications. These periods were defined by calendar years (1 January to 31 December) rather than by influenza epidemic weeks. The denominator therefore included the entire pediatric emergency department population, whereas influenza cases were restricted to laboratory-confirmed infections.

Statistical analysis was performed with the support of the Methodological Support Unit for Research. First, a descriptive analysis of the cohort characteristics was conducted. Qualitative variables were expressed as absolute and relative frequencies. Quantitative variables were described using measures of central tendency (mean and median), along with standard deviation and confidence intervals when appropriate. Normality of distribution was assessed using the Kolmogorov–Smirnov test. Quantitative variables were compared using Student’s *t*-test when normally distributed or the Mann–Whitney U test otherwise. Qualitative variables were compared using the chi-square test or Fisher’s exact test, as appropriate. A *p*-value ≤ 0.05 was considered statistically significant.

This study was conducted in accordance with the ethical principles of the Declaration of Helsinki (1975, revised in 2013). The study protocol was approved by the Ethics Committee of Hospital Clínico San Carlos (Approval Code: 24/840-E; Approval Date: 8 January 2025). Data were retrospectively collected from clinical records and handled in an anonymized and coded manner, ensuring the protection of participant identity and compliance with applicable personal data protection regulations. No information allowing direct or indirect patient identification was collected.

## 3. Results

A total of 617 episodes of influenza virus infection confirmed by polymerase chain reaction (PCR) or antigen testing were identified in pediatric patients aged 0 months to 18 years who attended the emergency department, pediatric wards, or pediatric intensive care unit. The mean age of the cohort was 4.7 ± 4.6 years, with a median of 3 years (IQR 1–6).

In terms of sex distribution, 352 patients (57.1%) were male and 265 (42.9%) were female.

**Figure 1:**
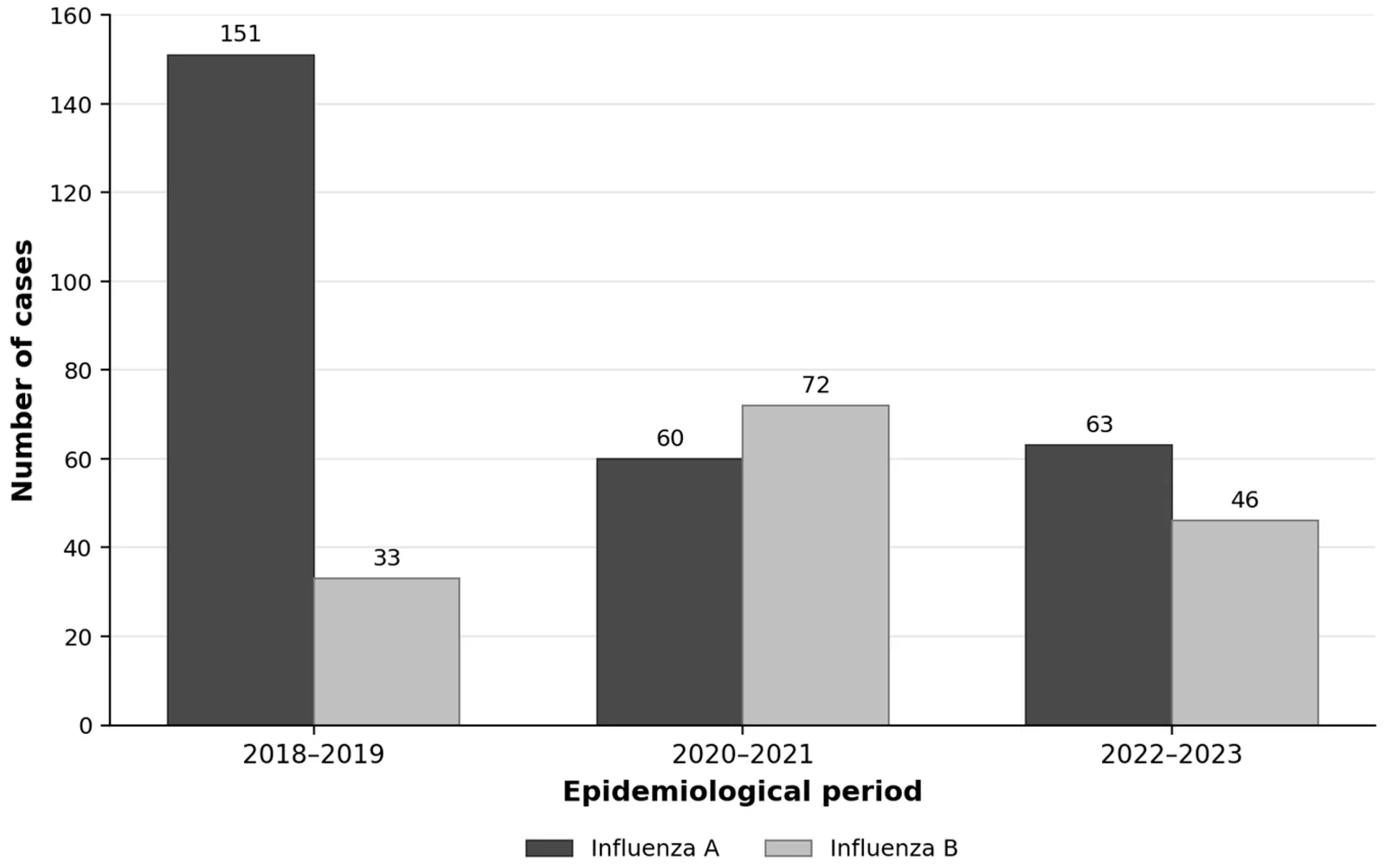
Distribution of influenza A and B among the three periods of study.

Regarding influenza virus type distribution across epidemiological periods, influenza A predominated during the pre-pandemic period (2018–2019), with 151 cases compared with 33 influenza B cases. During the pandemic period (2020–2021), influenza B predominated, with 72 cases compared with 60 influenza A cases. In the post-pandemic/transition period (2022–2023), influenza A again predominated, with 63 cases compared with 46 influenza B cases (Figure 1).

The most frequent clinical diagnosis was upper respiratory tract infection, present in 67.3% of cases, followed by pneumonia (13.8%) and bronchospasm/bronchiolitis (5.2%). The detailed distribution of clinical diagnoses is shown in Table 1.

A total of 108 patients (17.5%) had relevant medical history. The most frequent were otorhinolaryngological or respiratory conditions (50.0%). The remaining medical histories are detailed in Table 2. Notably, three patients had immunosuppressive conditions: two had controlled vertically transmitted HIV infection and one had autoimmune neutropenia.

Among the 158 hospitalized patients, 39 (24.7%) received oseltamivir for 5 days. Detailed information regarding the timing of antiviral initiation and the criteria used for treatment was not consistently available in the retrospective medical records; therefore, no reliable comparison between treated and untreated patients could be performed.

Two of the 158 admitted patients had received influenza vaccination in that season: one presented with acute encephalitis and the other with vomiting and inability to tolerate oral intake. Neither had relevant prior medical history. The retrospective records did not consistently contain sufficient information on the vaccine product or vaccination details, and the small number of vaccinated patients precluded a meaningful comparison of complications between vaccinated and unvaccinated patients.

Coinfections were documented in 42 of the 617 influenza episodes (6.8%), predominantly viral, while bacterial coinfection was observed in 2.6% of cases. The most frequently identified viral coinfections included respiratory syncytial virus (7 cases, 1.1%) and SARS-CoV-2 (4 cases, 0.6%). However, because microbiological testing was not performed using a uniform protocol across the cohort and was more frequently performed in hospitalized patients, reliable comparisons of clinical severity according to specific coinfecting pathogens could not be performed.

One death (0.2%) was recorded during the study period. This was a 3-year-old male with severe cerebral palsy who developed acute respiratory failure secondary to influenza A infection.

When analyzing the impact of the SARS-CoV-2 pandemic, three epidemiological periods were compared: pre-pandemic (2018–2019), pandemic (2020–2021), and the post-pandemic/transition period (2022–2023). Across these three periods, there were a total of 113 hospital admissions. The proportion of laboratory-confirmed influenza cases requiring hospital admission was 23.1% (57/247) in the pre-pandemic period, 16.5% (30/182) during the pandemic, and 14.8% (26/176) in the post-pandemic/transition period. When expressed relative to the total number of pediatric emergency visits, the corresponding admission rates were 0.23% (57/24,668), 0.16% (30/18,774), and 0.09% (26/27,507), respectively. The latter denominator was used to account for changes in the overall volume of pediatric emergency care across periods. Statistically significant differences were observed in admission rates across the three periods (*p* < 0.01; Table 3).

Considering the total number of pediatric emergency visits, the incidence of influenza was 1.0% (247/24,668) in 2018–2019, 0.97% (182/18,774) in 2020–2021, and 0.64% (176/27,507) in 2022–2023.

The mean age of patients with neurological complications was 6.4 ± 4.9 years, while that of patients with suspected acute viral myositis was 6.1 ± 1.5 years, compared with a mean age of 5.5 ± 5.2 years in the overall study cohort.

Neurological complications were identified in 33 patients (5.3%), and suspected acute viral myositis was identified in 23 patients (3.7%) in the overall study cohort. For the analysis of changes across epidemiological periods, Table 4 presents the number and incidence of neurological complications and suspected acute viral myositis during the three predefined periods (2018–2019, 2020–2021, and 2022–2023).

Among the 23 patients with suspected acute viral myositis, available CK levels ranged from 314 to 12,983 U/L, with a median value of 3252 U/L (IQR, 845–9306 U/L).

A higher proportion of neurological complications was observed in patients with influenza A compared with those with influenza B (5.5% vs. 5.0%), although this difference was not statistically significant (*p* = 0.78). In contrast, suspected acute viral myositis was significantly more frequent among patients with influenza B than among those with influenza A (7.3% vs. 1.8%; *p* < 0.01).

The frequency of neurological complications was higher among patients with underlying medical conditions than among those without underlying conditions (13.0% vs. 3.7%; *p* < 0.001). This association was particularly marked among patients with pre-existing neurological conditions, in whom neurological complications occurred in 31.6% compared with 3.6% among those without neurological comorbidities (*p* < 0.01). No statistically significant difference was observed in the frequency of suspected acute viral myositis between patients with and without underlying medical conditions (*p* = 0.16).

Seizures were the most frequent neurological complication, present in 66.7% of patients with neurological involvement. The most common type was generalized tonic–clonic seizures (40.9%). The distribution of seizure types and the presence of encephalitis are detailed in Table 5.

Regarding complementary investigations, 33.3% of patients with neurological complications underwent brain imaging, with magnetic resonance imaging being the most used modality. Only one study was abnormal, showing findings consistent with bacterial meningoencephalitis with subdural empyema secondary to frontal sinusitis caused by *Streptococcus intermedius*, in a patient with a concomitant diagnosis of influenza A infection.

Electroencephalography was performed in 18 of the 33 patients (54.5%), with abnormal findings in 9 cases (50%), consisting of generalized slowing or epileptiform activity. Lumbar puncture was performed in 5 patients: 3 were diagnosed with influenza-associated encephalitis, 1 with status epilepticus, and the last with bacterial meningoencephalitis secondary to frontal sinusitis with influenza coinfection, as previously described. Only the latter showed abnormalities in the cerebrospinal fluid, including hyperproteinorrachia, elevated nucleated cell count, and hypoglycorrhachia. PCR for herpes viruses was performed in all cerebrospinal fluid samples. PCR for influenza was not performed in cerebrospinal fluid. Therefore, encephalitis diagnoses were based on compatible clinical or electroencephalographic findings.

Finally, 78.8% of patients with neurological complications received treatment with antiepileptic drugs. A total of 15 patients (45.5%) were followed up in pediatric neurology clinics, with a favorable outcome in 80% of cases. Table 6 describes the 4 cases that required follow-up by pediatric neurology due to persistent neurological symptoms.

## 4. Discussion

This retrospective study analyzes a large pediatric cohort with confirmed influenza infection attended over seven consecutive seasons. Neurological complications and acute viral myositis were found to be relatively uncommon, although clinically highly relevant. The overall frequency of neurological complications was 5.3%, while myositis was identified in 3.7% of episodes—figures comparable to those reported in previous pediatric series, in which influenza-associated neurological involvement ranges between 4% and 10% of hospitalized patients, depending on the definition used and the characteristics of the cohort [1,2,3].

Regarding neurological complications, our results show that they represent a heterogeneous clinical spectrum, with seizures being the most frequent manifestation, present in approximately two-thirds of cases. This finding is consistent with previous observational studies and reviews, which identify seizures—both febrile and afebrile—as the most common neurological presentation associated with influenza infection in the pediatric population [2,3,10]. The relatively low proportion of encephalitis observed in our cohort, as well as the limited diagnostic yield of neuroimaging studies, supports the hypothesis that many of these neurological manifestations reflect a systemic inflammatory or metabolic mechanism rather than direct viral invasion of the central nervous system [3,4].

One of the most relevant findings of this study is the strong association between the presence of underlying neurological conditions and the development of neurological complications during influenza infection. Patients with pre-existing neurological disease showed a significantly higher frequency of neurological complications compared to those without such history. This result is consistent with multiple previous studies, which identify children with epilepsy, neurodevelopmental disorders, or structural neurological disease as a particularly vulnerable group for developing neurological complications in the context of systemic viral infections, including influenza [1,3,11]. It has been proposed that reduced neurological reserve and increased susceptibility to systemic inflammatory responses may explain this association [5,12]. As in other series, a considerable proportion of patients had not been vaccinated [2,3].

In contrast, no significant differences were observed in the frequency of neurological complications according to influenza virus type. This finding is consistent with other pediatric cohorts, in which no consistent increased neurological risk has been demonstrated for influenza A or B, suggesting that host-related factors play a more relevant role than virological characteristics in the development of these complications [2,4,11].

Regarding suspected acute viral myositis, our results show its significant association with influenza B infection, with a clearly higher frequency compared to influenza A cases. This association has been widely described in the literature and represents one of the classic features of benign acute childhood myositis [6,8,9,13]. As in previous studies, in our cohort, myositis followed a self-limited course and was not associated with prior medical history or greater overall clinical severity, reinforcing its generally benign nature [6,7,14,15].

Analysis by epidemiological periods showed that neither the frequency of neurological complications nor that of suspected acute myositis changed significantly during the pandemic or post-pandemic/transition periods. In Spain, national surveillance reported minimal influenza circulation during the 2020–2021 season, followed by a re-emergence of influenza activity during 2021–2022 [16,17]. The 182 laboratory-confirmed influenza episodes observed in our hospital-based dataset during the predefined 2020–2021 calendar period, despite the marked reduction in national influenza circulation, may therefore reflect the specific referral setting and testing patterns of our center rather than population-level influenza circulation. These findings suggest that, despite changes in viral circulation and healthcare utilization patterns resulting from the SARS-CoV-2 pandemic, the risk of these influenza-associated complications remained stable in our population. Similar findings have been reported in other recent series, in which no relevant variations in the neurological expression of influenza were observed after the pandemic [7].

In parallel, when considering the total number of pediatric emergency visits, a progressive decrease in the incidence of hospital admissions for influenza was observed across the three analyzed periods. This may reflect changes in the overall healthcare burden of influenza following the pandemic, although the explanation for this finding is unclear. In any case, no significant differences were detected in the proportion of neurological complications.

Coinfections were documented in a subset of patients, predominantly viral. However, because microbiological testing was not performed using a uniform protocol across the entire cohort and was more extensively performed among hospitalized patients, the frequency of documented coinfection may have been influenced by differences in diagnostic intensity. Therefore, no conclusions can be drawn regarding the association between coinfection and hospitalization or overall clinical severity. Previous studies have reported an association between increasing numbers of coinfecting pathogens and greater disease severity, whereas no significant trend was observed for encephalitis [18].

From a clinical perspective, the marked difference in the frequency of neurological complications between children with and without pre-existing neurological conditions (31.6% vs. 3.6%) identifies children with underlying neurological disorders as a particularly relevant subgroup for clinical surveillance during influenza infection [2,3,11]. In these patients, influenza may represent an important trigger for acute neurological decompensation, and clinicians should therefore consider a lower threshold for neurological evaluation when fever or respiratory symptoms are accompanied by changes in neurological status. Although most neurological manifestations in our cohort had a favorable outcome, the occurrence of persistent symptoms or the need for neurological follow-up in a subset of patients supports individualized follow-up according to the clinical presentation.

In contrast, the self-limited course of suspected acute viral myositis in our cohort supports conservative management with clinical monitoring and CK assessment when clinically indicated [8,9,14,15].

Our findings do not provide sufficient evidence to recommend changes in antiviral treatment strategies, particularly given the incomplete information regarding treatment initiation and the inability to reliably compare treated and untreated patients. This study has limitations inherent to its retrospective design, including possible underestimation of mild neurological manifestations and variability in the indication for complementary tests. Additionally, as a single-center study, generalization of the results should be approached with caution, particularly when considering the incidence of neuromuscular complications in the community or to secondary-care settings. In addition, blinded adjudication assessment was not performed. Another limitation is the missing information regarding the type of seizures. Distinguishing among febrile seizures, complex febrile seizures, and afebrile seizures would provide additional clinical information, although this information was not consistently available due to the retrospective design. Likewise, temporal association does not establish causality, and some events may have occurred coincidentally during influenza infection. With regard to complications frequency over time, the comparisons should be interpreted cautiously, due to the fact that differences in testing policies, viral circulation, and public health interventions may have influenced observed frequencies. Moreover, the relatively small number of complication events limits statistical power to detect modest differences. The absence of multivariable adjustment and formal power calculations represents an important limitation of the study.

However, its main strengths include the sample size, the long study period, and the simultaneous evaluation of neurological and muscular complications, providing a comprehensive view of the neuromuscular impact of influenza in the pediatric population.

A further limitation of the study concerns the definition of suspected acute viral myositis, which was based on compatible clinical findings and elevated CK levels, without systematic imaging or histopathological confirmation. In addition, CK measurements were not obtained according to a standardized serial protocol, particularly in patients who were not admitted, limiting the assessment of peak CK levels and the possibility of reliably classifying cases according to the severity of CK elevation.

## 5. Conclusions

In this pediatric cohort, neurological complications and suspected acute myositis associated with influenza infection were infrequent but clinically relevant. Neurological complications were not associated with influenza virus type or epidemiological period, whereas suspected myositis was more frequent in influenza B infections but did not differ significantly across epidemiological periods. The presence of underlying neurological conditions was identified as the main factor associated with the development of neurological complications. These findings highlight the importance of close clinical monitoring in children with neurological comorbidities and provide useful information for risk stratification in pediatric patients with influenza infection.

## Figures and Tables

**Table 1:** Clinical diagnoses among pediatric patients with laboratory-confirmed influenza infection (*n* = 617).

Diagnosis	Absolute Value	Percentage (%)
Upper respiratory tract infection	415	67.3
Pneumonia	85	13.8
Bronchospasm/bronchiolitis	32	5.2
Acute gastroenteritis	16	2.6
Seizures	19	3.1
Suspected acute viral myositis	23	3.7
Encephalitis/meningitis	5	0.8
Other diagnoses	18	2.9
Death	1	0.2

**Table 2:** Distribution of relevant underlying medical conditions among pediatric patients with laboratory-confirmed influenza infection and a documented medical history (*n* = 108).

Underlying Medical Condition	N	%
Otorhinolaryngological/respiratory disease	54	50.0
Febrile seizures/epilepsy	17	15.7
Neurodevelopmental delay or congenital neurological disease	14	13.0
Prematurity	5	4.6
Heart disease	5	4.6
Headache/migraine	4	3.7
Hemoglobinopathies (sickle cell disease)	3	2.8
Previous encephalitis/meningitis	3	2.8
Immunosuppression	3	2.8

**Table 3:** Hospital admissions and admission rates among pediatric patients with laboratory-confirmed influenza according to epidemiological period.

Epidemiological Period	Laboratory-Confirmed Influenza Cases (*n*)	Influenza Admissions, *n* (%)	Pediatric Emergency Visits, *n*	Hospital Admission Among Emergency Visits, %	*p*-Value
2018–2019	247	57	24,668	0.23	
2020–2021	182	30	18,774	0.16	
2022–2023	176	26	27,507	0.09	<0.01

**Table 4:** Frequency of neurological complications and suspected acute viral myositis among pediatric patients with laboratory-confirmed influenza according to epidemiological period.

	2018–2019 (*n*, %)	2020–2021 (*n*, %)	2022–2023 (*n*, %)	*p*-Value
Neurological complications	9 (0.036)	6 (0.03)	7 (0.03)	0.811
Myositis	5 (0.02)	10 (0.05)	7 (0.03)	0.168

**Table 5:** Distribution of neurological complications among pediatric patients with laboratory-confirmed influenza and neurological involvement (*n* = 33).

Neurological Manifestation	*n* (%)
Seizures	
No	11 (33.3)
Yes	22 (66.7%)
Type of seizure	
Typical febrile seizure	3 (13.6)
Atypical febrile seizure	1 (4.5)
Status epilepticus	4 (18.2)
Myoclonus	1 (4.5)
Catatonia	2 (9.1)
Generalized tonic–clonic seizure	9 (40.9)
Absence seizure	1 (4.5)
Encephalitis	
No	28 (84.4)
Yes	5 (15.2)

Percentages for seizure types are calculated among patients with seizures (*n* = 22), whereas percentages for encephalitis are calculated among all patients with neurological complications (*n* = 33).

**Table 6:** Clinical characteristics and outcomes of pediatric patients with persistent neurological symptoms following influenza-associated neurological complications (*n* = 4).

Case	Age (Years)	Sex	Year	Influenza Type	Underlying Neurological Condition	Diagnosis	Admission	Follow-Up
A	3	Female	2018	A	Dravet syndrome	Pneumonia and febrile seizures	Yes, PICU	Dravet syndrome. Follow-up in another center
B	1	Female	2020	A	No	Atypical febrile seizure	Yes, ward	Atypical febrile seizures (status epilepticus). Required AED
C	9	Female	2022	A	No	Non-epileptic paroxysmal event	Yes, ward	Sleep myoclonus and night terrors
D	3	Female	2023	A	Dravet syndrome	Respiratory infection and febrile seizures	Yes, ward	Dravet syndrome. Required AED

AED: antiepileptic drug. PICU: pediatric intensive care unit.

## Data Availability

The data that support the findings of this study are available from the corresponding author upon reasonable request.
